# Risk factors and clinical correlates of neoplastic transformation in gastric hyperplastic polyps in Chinese patients

**DOI:** 10.1038/s41598-020-58900-z

**Published:** 2020-02-13

**Authors:** Haiyi Hu, Qian Zhang, Guangyong Chen, D. Mark Pritchard, Shutian Zhang

**Affiliations:** 1 Department of Gastroenterology, Beijing Friendship Hospital, Capital Medical University; National Clinical Research Center for Digestive Diseases; Beijing Digestive Disease Center; Beijing Key Laboratory for Precancerous Lesion of Digestive Diseases, Beijing, 100050 China; 2 Clinical Epidemiology and EBM Unit, Beijing Friendship Hospital, Capital Medical University; National Clinical Research Center for Digestive Disease, Beijing, 100050 China; 3 Department of Pathology, Beijing Friendship Hospital, Capital Medical University; National Clinical Research Center for Digestive Diseases, Beijing, 100050 China; 4 Gastroenterology Research Unit, Department of Cellular and Molecular Physiology, Institute of Translational Medicine, University of Liverpool, Liverpool, L69 3GE UK

**Keywords:** Gastric cancer, Gastritis, Gastrointestinal hormones

## Abstract

Gastric hyperplastic polyps (GHPs) have a potential risk of neoplastic transformation, but the responsible mechanisms have not yet been established. We conducted a study involving 55 patients (33 female) who had undergone endoscopic or surgical resection of GHPs. We compared 16 patients who had GHPs showing neoplastic transformation with 39 patients who had non-neoplastic GHPs. We analyzed differences in serology, gastroscopic manifestations and pathology between the two groups in order to establish risk factors that may be associated with neoplastic transformation. The mean age of the cohort was 61.73 ± 9.024 years. The prevalence of positive serum gastric parietal cell antibody (PCA) was 61.8%. 30 of the GHPs with neoplastic formation had a “strawberry-like” appearance with erosions of polyps (*P* = 0.000). A history of anaemia was a risk factor for GHPs which demonstrated neoplastic transformation (odds ratio [OR], 3.729; 95% confidence interval [CI], 1.099–12.649; *P* = 0.035). Although the differences were not significant, our data showed higher prevalences of positive serum PCA (*P* = 0.057), hypergastrinemia (*P* = 0.062) and female gender (*P* = 0.146) in the GHP patients who had neoplastic transformation. Multiple polyps in the corpus (*P* = 0.024) occurred more frequently in serum *P*CA positive patients. Hypergastrinemia occurred more frequently in *Helicobacter pylori* negative patients and of these 20/22 patients had a positive PCA (*P* = 0.007). GHPs are associated with autoimmune metaplastic atrophic gastritis (AMAG). AMAG is probably one of the risk factors for GHPs to undergo neoplastic transformation.

## Introduction

Gastric hyperplastic polyps (GHPs) are one of the most common type of polyp encountered in the stomach^1^. They constitute 30–93% of all benign epithelial gastric polyps^2–4^ and occur in patients of either gender and are more common with increasing age (65 to 70 years on average)^5^. GHPs are usually asymptomatic and are often diagnosed incidentally during routine esophagogastroduodenoscopy (EGD). However they may cause anaemia. GHPs can be sessile or pedunculated and can occur as single or multiple polyps in the antrum or corpus of the stomach. The diameter of GHPs is usually less than 1 cm, however 10% measure greater than 2 cm^6^. GHPs commonly arise within a background of chronic gastritis, including *Helicobacter pylori*-related gastritis (type B gastritis), as well as autoimmune metaplastic atrophic gastritis (AMAG, type A gastritis)^7^. Up to 70–80% of GHPs have been found to regress after eradication of *Helicobacter pylori* and these polyps do not need to be removed endoscopically^8,9^.

GHPs have a potential risk of neoplastic transformation, so they have been classified as potentially precancerous lesions. With the recent more widespread use of endoscopic resection of gastric polyps, an increased number of cases of dysplasia or carcinoma within GHPs has been reported^10^. The responsible mechanisms however remain unknown. This study aimed to analyze possible risk factors that are associated with the neoplastic transformation of gastric hyperplastic polyps.

## Results

### Demographics features

During January 2010 and June 2017, there were 6418 cases of gastric polyp diagnosed. 1115 of these (17.4%, 1115/6418) were histologically confirmed GHPs. 30 of these GHPs (2.69%, 30/1115) were diagnosed as containing dysplasia or adenocarcinoma. The mean age was 61.73 ± 9.024 years old (range 32–79). Absence of *Helicobacter pylori* was found in 63.6% (35/55). Within the 16 patients who showed evidence of neoplastic transformation there were 12 females and 4 males, the mean age was 63.44 ± 12.27 years (range 32–79). 9 (56.3%) of the patients who had GHPs with neoplastic transformation and 10 (25.6%) of the patients with pure GHPs had a history of anaemia. The patients who had a history of anaemia were more likely to have GHPs with evidence of neoplastic transformation (9/16 vs 10/39 in the GHP patients with or without neoplastic transformation, χ^2^ = 4.701, *P* = 0.030) (Table 1).

**Table 1:** Demographics features of GHP patients.

Parameters mean ± SD; n (%)	Total (n = 55)	GHP patients with neoplastic formation (n = 16)	GHP patients without neoplastic formation (n = 39)	*P* value
Age (years)	61.73 ± 9.02	63.44 ± 12.27	61.03 ± 7.39	0.373
BMI(Kg/m^2^)	24.7 ± 4.14	25.88 ± 4.75	24.22 ± 3.82	0.179
Gender				0.146
Male	22 (40.0)	4 (25.0)	18 (46.2)	
Female	33 (60.0)	12 (75.0)	21 (53.8)	
*H. pylori* infection				0.262
Yes	20 (36.4)	4 (25.0)	16 (41.0)	
No	35 (63.6)	12 (75.0)	23 (59.0)	
History of anaemia				0.030
Yes	19 (34.5)	9 (56.3)	10 (25.6)	
No	36 (65.5)	7 (43.8)	29 (74.4)	

The history of anaemia showed significant difference between GHP patients with and without neoplastic formation, tested by Chi-square test (*P* = 0.030). GHP, gastric hyperplastic polyp; BMI, body mass index; SD, standard error; *H. pylori, Helicobacter pylori*.

The initial indications for performing EGD were: 27 patients screening for early gastric cancer, 15 bloating, 4 abdominal pain, 7 heartburn or acid reflux and 2 anaemia. However, 20 patients (36.4%) actually had evidence of anaemia when tested. Only 3 patients took PPI, but all of them had normal fasting serum gastrin-17 concentrations.

### Serum tests in GHP patients with or without neoplastic transformation

The positive rate of serum anti-gastric parietal cell antibody (PCA) was 61.8% (34/55), 13/16 vs 21/39 in GHP patients with or without neoplastic transformation (χ^2^ = 3.610, *P* = 0.057). Only 6 patients (6/55) had positive anti-intrinsic factor antibodies. Hypergastrinemia occurred in 27 patients, 11 of whom were GHP patients with neoplastic transformation (11/16, χ^2^ = 3.489, *P* = 0.062). 20 patients had anaemia, 9/16 vs 11/39 in GHPs with or without neoplastic transformation (χ^2^ = 3.856, *P* = 0.05). There were 33 females in the study, 12/16 vs 21/39 in GHPs with or without neoplastic transformation (χ^2^ = 2.115, *P* = 0.146). Although, there were no significant differences in terms of positive PCA, hypergastrinemia or gender, there appeared to be trends in these parameters in those patients who had GHPs with neoplastic transformation (Table 2).

**Table 2:** Serum tests results in GHP patients with or without neoplastic transformation.

Parameters n (%)	Total (n = 55)	GHP patients with neoplastic formation (n = 16)	GHP patients without neoplastic formation (n = 39)	*P* value
Hypergastrinemia				0.062
Yes	27 (49.1)	11 (68.8)	16 (41.0)	
No	28 (50.9)	5 (31.3)	23 (59.0)	
PCA				0.057
Positive	34 (61.8)	13 (81.3)	21 (53.8)	
Negative	21 (38.2)	3 (18.8)	18 (46.2)	
IF				0.660
Positive	6 (10.9)	1 (6.3)	5 (12.8)	
Negative	49 (89.1)	15 (93.8)	34 (87.2)	
Anaemia				0.050
Yes	20 (36.4)	9 (56.3)	11 (28.2)	
No	35 (63.6)	7 (43.8)	28 (71.8)	
Ferritin				0.629
Low	18 (32.7)	6 (37.5)	12 (30.8)	
Normal	37 (67.3)	10 (62.5)	27 (69.2)	
Hypothyroidism				0.258
Yes	9 (16.4)	1 (6.3)	8 (20.5)	
No	46 (83.6)	15 (93.8)	31 (79.5)	

PCA, serum anti-parietal cell antibody; IF, anti-intrinsic factor antibody.

### Endoscopic characteristics of GHPs with or without neoplastic transformation

There were 141 GHPs in total in the 55 patients, including 31 GHPs which had evidence of neoplastic transformation (the locations were: fundus 6, corpus 20 and antrum 5) and 110 pure GHPs (located in: fundus 27, corpus 58 and antrum 25). 30 of the GHPs with neoplastic formation had a “strawberry-like” appearance with erosions at the head of polyps, 30/31 vs 67/110 in GHPs with or without neoplastic transformation (χ^2^ = 14.491, *P* = 0.000)(Table 3). Polyps occurring in the patients who had positive serum PCA were more frequently found in the fundus and corpus than the antrum (90/111 vs 17/30 in the fundus and corpus vs antrum, χ^2^ = 7.693, *P* = 0.006).

**Table 3:** Endoscopic features of GHPs with or without neoplastic formation.

Parameters n (%)	GHPs with neoplastic formation(n = 31)	GHPs without neoplastic formation(n = 110)	P value
“Strawberry-like” with erosions			< 0.001
Yes	30(96.8)	67(60.9)	
No	1(3.2)	43(39.1)	
Morphology			0.124
Pedunculated	26(83.9)	77(70)	
Sessile	5(16.1)	33(30)	
Size			0.373
≥1 cm	26(83.9)	84(76.4)	
<1 cm	5(16.1)	26(23.6)	

The “Strawberry-like” with erosions showed significant difference between GHPs with and without neoplastic formation, tested by Chi-square test (*P* < 0.001).

### The characteristics of GHP patients who had anaemia

20 (20/55) patients had anaemia, of whom 16 patients had iron deficiency (16/20 vs 4/35 in patients with or without anaemia, χ^2^ = 25.861, *P* = 0.000), 6 had vitamin B12 deficiency and 3 patients had both iron and vitamin B12 deficiency. Women were four times more likely than men to be anaemic (16/20 vs 17/35 in patients with or without anaemia, χ^2^ = 5.238, *P* = 0.022). 19 patients had a history of anaemia, of whom 17 patients had current anaemia and 16 patients had a positive PCA.

### The characteristics of GHP patients who had evidence of helicobacter pylori infection

20 (20/55) patients had evidence of *Helicobacter pylori* infection. Interestingly, hypergastrinemia occurred more frequently in the patients who did not have *H. pylori* infection (5/20 vs 22/35 in GHPs with or without *H. pylori* infection, χ^2^ = 7.299, *P* = 0.007).

### Pathological characteristics of GHPs with or without neoplastic transformation

Seven patients had adenocarcinoma (6 females, 1 male) of which 5 were well-differentiated and 2 were moderately-differentiated cancers. Three female patients had GHPs that contained foci of high grade dysplasia and 6 patients had GHPs containing areas of low grade dysplasia (3 females, 3 males). No metastases were found in any patients. However only 3 of these patients had been diagnosed with GHPs that showed neoplastic transformation by initial forceps biopsy prior to endoscopic resection. 13 out of the 16 GHPs with neoplastic transformation were only diagnosed following EMR or ESD, so the severity of disease was underestimated before resection in 81.3% (13/16) of these patients.

Evaluation of the background mucosa was undertaken in 14 of the patients who had GHPs with neoplastic transformation. 10 of these patients were diagnosed with autoimmune metaplastic atrophic gastritis (AMAG) or enterochromaffin-like cell (ECL-cell) hyperplasia (all had positive serum PCA); 3 patients had intestinal metaplasia in the corpus (2 had positive serum PCA) and 1 patient had chronic gastritis and positive serum PCA. The pathological characteristics of the GHPs which had neoplastic transformation are recorded in Table 4.

**Table 4:** Summary of pathological data on patients with GHPs demonstrating neoplastic transformation.

No.	Gen-der	Age	Seru-m PCA	*H. p* status	Size	Single(S)/Multiple (M) (location)	Location of neoplastic polyps	Mor-phol-ogy	Forceps biopsy histology	Background mucosa	Pathology of EMR/ESD/Surgery
1	F	69	+	−	>2 cm	M(C)	C	S	Gastritis with IM	AMAG/ECL-cell hyperplasia	HGD
2	F	77	+	−	>2 cm	M(F + C + A)	F + C + A	P	GHP with HGD	AMAG	Well-differentiated adenocarcinoma (17 polyps,10/17 polyps with carcinoma)
3	F	59	+	−	>1 cm	M (C + A)	C	P	GHP	AMAG/ECL-cell hyperplasia	Well-differentiated adenocarcinoma
4	F	66	+	−	>2 cm	M (C + A)	C + A	P	GHP	AMAG/ECL-cell hyperplasia	Moderately-differentiated adenocarcinoma /HGD (Surgery: lv invasion, Ki-67:70%)
5	F	59	+	+	>1 cm	M(C)	C	P	GHP with HGD	ECL-cell hyperplasia	Well-differentiated adenocarcinoma
6	F	78	+	−	>1 cm	S(F)	F	P	GHP	AMAG	Well-differentiated adenocarcinoma
7	F	65	+	−	>1 cm	S(A)	A	P	Gastritis with IM	AMAG/ECL-cell hyperplasia	HGD
8	F	44	−	−	>1 cm	S(Cardia)	Cardia	S	GHP	ND	LGD
9	F	63	+	−	>1 cm	S(A)	A	P	GHP	Chronic gastritis with IM	LGD
10	F	58	+	−	>1 cm	M(F + C)	F	P	Gastritis	AMAG/ECL-cell hyperplasia	HGD
11	F	32	−	+	>1 cm	S(C)	C	P	GHP	ND	LGD
12	F	79	+	−	>1 cm	M(F + C)	F + C	P	GHP with HGD	AMAG/ECL-cell hyperplasia	Moderately-differentiated adenocarcinoma/HGD
13	M	74	−	−	>2 cm	M (C + cardia)	C	P	Gastritis with IM	IM/tubular adenoma in antrum	LGD
14	M	64	+	+	<1 cm	M (C + A)	C	S	GHP	IM	LGD
15	M	68	+	+	>2 cm	S(C)	C	P	GHP	Chronic gastritis	LGD
16	M	60	+	−	>2 cm	M(C)	C	P	Gastritis with IM	AMAG/ECL-cell hyperplasia	Well-differentiated adenocarcinoma

M, male; F, female; M, multiple; S, single; F, fundus; C, corpus; A, antrum; P, pedunculated; S, sessile; IM, intestinal metaplasia; AMAG, autoimmune metaplastic atrophic gastritis; ECL-cell, enterochromaffin-like cell; HGD, high grade dysplasia; LGD, low grade dysplasia; lv, lymphovascular; ND, not done.

### Potential risk factors for GHPs with or without neoplastic transformation

Univariate and multivariate logistic regression analyzes was performed to explore potential associations between risk factors and the presence of neoplastic transformation in GHPs. Continuous variables were transformed into grade variables namely age (<60 years, ≥60 years) and BMI (<24 kg/m^2^, ≥24 kg/m^2^). In univariate logistic regression analysis, history of anaemia was significantly associated with GHPs that showed neoplastic transformation (odds ratio [OR], 3.729; 95% confidence interval [CI], 1.099–12.649; *P* = 0.035). The other factors tested were not significantly associated with GHPs that demonstrated neoplastic transformation (all *p* values ≥0.05) (Table 5). In addition, when history of anaemia, hypergastrinemia, ferritin, PCA and anaemia were added into a multivariate logistic regression model, the results still showed that a history of anaemia was significantly associated with GHPs that demonstrated neoplastic transformation (OR = 5.603; 95% CI: 1.167–26.899; *P* = 0.031).

**Table 5:** Assessment of risk factors for GHPs with neoplastic transformation in univariate logistic regression.

Variables	95%CI	OR	*P* value
Age (<60 years vs. ≥60years)	0.291–3.595	1.023	0.972
BMI (<24 kg/m^2^ vs. ≥24 kg/m^2^)	0.140–1.636	0.478	0.240
Gender (Male vs. Female)	0.107–1.420	0.389	0.153
*H*. *pylori* infection (Yes vs. No)	0.131–1.757	0.479	0.267
Hypergastrinemia (Yes vs. No)	0.920–10.871	3.162	0.068
PCA (Postive vs. Negative)	0.912–15.129	3.714	0.067
IF (Postive vs. Negative)	0.049–4.222	0.453	0.487
Anaemia (Yes vs. No)	0.977–10.966	3.273	0.055
Serum iron (Low vs. Normal)	0.683–7.417	2.250	0.183
Vit B_12_ (Low vs. Normal)	0.377–6.156	1.524	0.554
Ferritin (Low vs. Normal)	0.399–4.570	1.350	0.630
Hypothyroidism (Yes vs. No)	0.030–2.259	0.258	0.221
History of anaemia (Yes vs. No)	1.099–12.649	3.729	0.035

GHPs patients who had history of anaemia showed higher risk of neoplastic transformation than GHPs patients who did not have history of anaemia (OR = 3.729, 95%CI: 1.099–12.649, *P* = 0.035). OR, odds ratio; CI, confidence interval.

## Discussion

GHPs used to be the most common polyps of benign epithelial gastric polyps^1^. However, the prevalence of GHPs has decreased to 14–40% of gastric polyps in recent decades^11–13^. In our study of 6418 cases of gastric polyp, 1115 cases (17.4%, 1115/6418) were GHPs. These data are similar to those reported in US^14^. A Japanese study reported that all GHPs were more common in females, but that the sex ratio of GHPs that contained carcinoma was 1:1^15^. In our study, female gender was slightly preponderant overall (F:M = 1.5:1), and this was especially in the patients who had GHPs with neoplastic transformation (F:M = 3:1). To date, dysplasia or adenocarcinoma has been infrequently described in GHPs, and these polyps are therefore generally interpreted as being benign lesions which have a malignant potential but also act as markers for the precancerous condition of the gastric mucosa^16^. The reported prevalence of dysplasia in GHPs has varied from 1.9–19%^17,18^; the frequency of infiltrating adenocarcinoma arising in GHPs has been debated but has ranged from 0–13.5%^19,20^. In our study 2.69% (30/1115) of patients who were diagnosed with GHPs showed evidence of dysplasia or adenocarcinoma within these polyps.

In this study we noticed a phenomenon of an unusual environment in which GHP neoplastic transformation may occur: namely an association between neoplastic GHPs and the presence of AMAG in the background gastric mucosa. Some GHPs showed evidence of focal neuroendocrine differentiation or ECL-cell hyperplasia in the gastric mucosa surrounding the tumor. Whether the presence of AMAG may constitute an additional risk factor for neoplastic change within GHPs remains uncertain. Some case reports have described the probable relationship between GHPs and dysplasia or carcinoma^21–23^. Neuroendocrine differentiation occurs in 39.6% of gastric cancer cases and occurs more frequently in poorly differentiated cancers than in well differentiated tumors^24^.

The approximate annual detection rate of AMAG reported recently by Hejun Zhang *et al*. in China is about 0.9%^25^, which is similar to the incidence in Asians (1.4%) reported by Park *et al*.^26^. Patients with AMAG are more likely to develop gastric polyps and neoplasms. In Park’s study, 143 of 461 (31%) patients with AMAG had gastric lesions: 138 of these were GHPs, 21 were adenomas and 11 were adenocarcinomas. The importance of GHPs appears to lie most significantly in their status as a marker for an abnormal background gastric mucosa rather than as isolated pre-neoplastic lesions. GHPs arise most commonly in a background of abnormal and often atrophic gastric mucosa. In Abraham’s study^27^, the true rate of a normal or only mildly abnormal gastric mucosal background in patients with GHPs was probably lower than 15%. The data demonstrated that 25% GHPs arose in the setting of active chronic *H*. *pylori* gastritis, 21% arose in association with reactive/chemical gastropathy, and 12% arose in autoimmune gastritis. According to Orlowska *et al*.^28^, the risk of malignancy developing in the gastric mucosa outside the polyp is slightly higher than that within the polyp itself. Markowski *et al*. also showed that the chance of malignant development in the surrounding mucosa was 7.1%, whereas within the polyp itself it was 2.1%^29^. The risk of gastric cancer developing in subjects with severe fundal atrophic gastritis was 5.76 times greater than in those having little or no fundal atrophic gastritis^30^. Pathologists diagnosing GHP in patients who have not had adequate biopsies of the non-polypoid stomach should therefore reiterate this association and recommend that biopsies of the surrounding mucosa be taken to evaluate any underlying gastric abnormalities. In addition, as we know, atrophic gastritis and intestinal metaplasia are the major precursor lesions of gastric adenocarcinoma. However, a Japanese study suggested that intestinal metaplasia within GHPs was not associated with carcinogenesis within these polyps. They suggested that GHPs with neoplastic changes develop via a hyperplasia-dysplasia-carcinoma sequence rather than an intestinal metaplasia-dysplasia-carcinoma sequence^20^.

It is well known that GHPs are commonly associated with *H. pylori* infection, and GHPs regress in the great majority of cases following eradication of this infection. In a large cohort study by Nam *et al*., *H. pylori* infection was associated with a 2-fold increased risk of diagnosing GHPs, while successful *H. pylori* eradication and persistent *H. pylori* negative status resulted in an 11.7- fold increase in the clearance of GHPs compared to control^31^. Some patients with *H. pylori* infection may have hypergastrinemia, but in this study, hypergastrinemia occurred more frequently in the patients who did not have *H*. *pylori* infection. This is probably because most of the *H*. *pylori* negative patients (30/55) had positive PCAs and thus an alternative cause of hypochlorhydria and hypergastrinemia.

Interestingly, the patients who had positive PCA were more likely to have iron deficiency anaemia than vitamin B_12_ deficiency. This is probably because the progression of AMAG to pernicious anaemia (PA) is likely to take 20–30 years^32^, PA with vitamin B_12_ deficiency occurs in the later stages of AMAG. The incidence of gastric hyperplastic polyps in iron-deficient patients was reported to be 1.4%^33^. In our study, logistic regression analysis showed that a history of anaemia was a significant risk factor for GHPs that showed neoplastic transformation. This has not been reported in previous studies. 19 patients had a history of anaemia, of whom 17/19 had current anaemia and 16/19 had a positive PCA. Whether or not this means that long term anaemia correlates with the presence of AMAG remains speculative however.

In conclusion, GHPs are associated with the presence of AMAG, and this may be responsible for neoplastic transformation in some cases, and hypergastrinemia may contribute. Our study suggests that a history of anaemia is more likely to have GHPs which develop neoplastic transformation. The main limitation of our study was the small sample size which has reduced its statistical power to investigate whether or not the coexistence of GHP and AMAG (or ECL-cell hyperplasia) confers an increased risk of developing dysplasia and carcinoma. However, additional research on this topic is urgently needed.

## Methods

The hospital based study was conducted at the Beijing Friendship Hospital, Capital Medical University, China. During January 2010 and June 2017. There were 6418 cases of gastric polyp diagnosed. 1115 patients were histologically confirmed GHPs, 701 female patients and 414 male patients. 30 patients who had GHPs that demonstrated neoplastic transformation including adenocarcinoma, high grade dysplasia and low grade dysplasia were diagnosed. 16 of these GHP patients agreed to participate in this study and therefore underwent additional evaluations. 60 GHP patients who didn’t show neoplastic transformation were randomly selected from 1085 patients and 39 of these patients were agreed to participate in this study. We finally succeeded in reviewing 16 GHP patients who had neoplastic transformation and 39 GHP patients who had no evidence of neoplastic transformation (subsequently referred to as pure GHPs). We analyzed the difference in the participation rate between the two groups (53% vs 65%), but this showed no significance, with P value of 0.285. We excluded patients who had polyposis syndromes such as Juvenile polyposis, Peutz-Jeghers syndrome, Cronkhite-Canada syndrome. Informed consent was obtained from all individual participants included in the study. The study was approved by Beijing Friendship Hospital Ethics Committee (Certificate number is 2017-P2-110-01) and registered in the Chinese Clinical Trial Registry, World Health Organization (Registered number is ChiCTR1800014493).

All 55 patients had blood taken to measure their fasting serum gastrin-17 concentration (normal range 35–100 pg/ml, ELISA), serum anti-parietal cell antibody (PCA, abnormal >1:80, IIFA), serum anti-intrinsic factor antibody (IF, abnormal >1:20, ELISA), serum thyroid function tests (TFTs), hemoglobin (male 120–160 g/l, female 110–150 g/l), serum vitamin B12 (abnormal <190 pg/ml), serum iron (normal range 7.8–32.2 umol/l), serum ferritin (normal range 11.0–306.0 ng/ml) and *H. pylori* serology. These hematological and biochemical tests were undertaken using standard laboratory procedures. *H. pylori* status was determined by serum *H. pylori* IgG and ^13^C-urea breath test (UBT) or by histological evaluation of gastric biopsies. All 55 patients had a serum *H. pylori* IgG test. 44 additionally underwent UBT and 4 patients had histological evaluation. As long as one test result was positive, the final result was deemed positive. In patients in whom both the serum IgG test and UBT were negative, the final result was deemed negative. *H. pylori* negative patients did not have any prior eradication therapy or receive any proton pump inhibitor / H_2_ receptor antagonist before the test. Hypergastrinemia was diagnosed when the fasting serum gastrin concentration was at least two times higher than the upper limit of the normal range.

**Figure 1:**
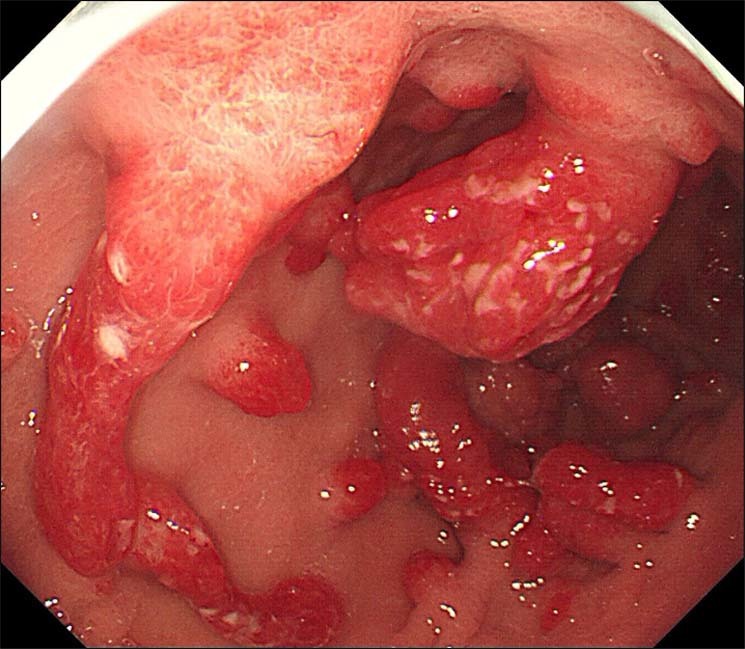
WLI shows multiple GHPs with neoplasia in the corpus, ‘strawberry-like’ with erosions on the surface. This original image was processed in photoshop for changing to 300 bpi.

All the GHPs were treated by endoscopic mucosal resection (EMR), endoscopic submucosal dissection (ESD) or surgery. White light imaging (WLI) gastroscopy was performed using Olympus GIF H260 endoscopes. EMR or ESD was performed using GIF Q260J endoscopes. Using WLI, GHPs normally demonstrate a strong reddish coloration; they are usually semispherical when small or semi-pedunculated to pedunculated when they become larger. We describe the appearance as “strawberry-like” polyps. When GHPs harbor dysplasia or adenocarcinoma, this always appeared to be associated with surface erosion, especially with dirty or purulent ulcers being seen on the surface of the polyps (Fig. 1).

Histological slides were re-evaluated by an experienced pathologist according to the WHO Classification of Tumours of the Digestive System. We used the updated Sydney System to diagnose atrophic gastritis and the OLGA (Operative Link on Gastritis Assessment) system to evaluate the risk of gastric cancer^34–36^. The background biopsies were taken from the antrum and corpus/fundus, with two endoscopic bites being taken at each site. Elevated fasting serum gastrin concentrations and the presence of anti-parietal cell antibody or anti-intrinsic factor antibodies were also used to corroborate the diagnosis of AMAG^24^. The detailed demographic features and OLGA stages of the GHPs with neoplastic transformation were showed in Supplementary Table S1.

Statistical analysis was performed using SPSS 22. Quantitative variables were described and analyzed using the mean ± SD and student t test, whereas categorical variables were described using frequency and proportions. The Chi-square test was used to test for differences between groups. Fisher’s exact test was used for statistical comparisons as appropriate. Univariate and multivariate logistic regression analysis was performed to explore the association of risk factors. *P* values < 0.05 were considered statistically significant. All *p* values were two-sided.

### Ethical statement

All procedures performed in studies involving human participants were in accordance with the ethical standards of the institutional and/or national research committee and with the 1964 Helsinki declaration and its later amendments or comparable ethical standards.

## Supplementary information

Supplementary information .

## Data Availability

The datasets generated and analyzed during the current study are available from the corresponding author on reasonable request.
