# Supplementary material for: Risk factors and clinical correlates of neoplastic transformation in gastric hyperplastic polyps in Chinese patients
Source: Sci Rep. 2020 Feb 13;10:2582. doi: 10.1038/s41598-020-58900-z (PMC7018716; doi:10.1038/s41598-020-58900-z)
Supplement: Supplementary file 1 — Supplementary information . [file 41598_2020_58900_MOESM1_ESM.docx]

**Risk factors and clinical correlates of neoplastic transformation in gastric hyperplastic polyps in Chinese patients**

Haiyi Hu^1^, Qian Zhang^2^, Guangyong Chen^3^, D. Mark Pritchard^4^, Shutian Zhang^1*^

^1^ Department of Gastroenterology, Beijing Friendship Hospital, Capital Medical University; National Clinical Research Center for Digestive Diseases; Beijing Digestive Disease Center; Beijing Key Laboratory for Precancerous Lesion of Digestive Diseases, Beijing, 100050, China

^2^ Clinical Epidemiology and EBM Unit, Beijing Friendship Hospital, Capital Medical University; National Clinical Research Center for Digestive Disease, Beijing, 100050, China

^3^ Department of Pathology, Beijing Friendship Hospital, Capital Medical University; National Clinical Research Center for Digestive Diseases, Beijing, 100050, China

^4^ Gastroenterology Research Unit, Department of Cellular and Molecular Physiology, Institute of Translational Medicine, University of Liverpool, Liverpool, L69 3GE, UK

***Corresponding author：**Professor Shutian Zhang

|  | Stage 0 | Stage I | Stage II | Stage III | Stage IV |
| --- | --- | --- | --- | --- | --- |
| Cases (n=14) | 0 | 1 | 4 | 5 | 4 |
| Male/Female (n/n) | 0 | 1/0 | 1/3 | 1/4 | 0/4 |
| H*. p* +/- (n/n) | 0 | 1/0 | 1/3 | 1/4 | 0/4 |
| Neoplastic lesions | 0 | 1LGD | 3LGD/ 1 HGD | 2HGD/ 3AC | 4 AC |

Supplementary Table S1. Demographic feature and OLGA stages of the GHP with neoplastic transformation.

Using the updated Sydney System to diagnose atrophic gastritis and the OLGA system to evaluate the risk of

gastric cancer. H*. p:* Helicobacter pylori; LGD: low-grade dysplasia; HGD: high-grade dysplasia; AC: adenocarcinoma; OLGA: Operative Link on Gastritis Assessment
